# Case Report: Decompressive Craniectomy for COVID-19 Malignant Cerebral Artery Infarction. Is Surgery a Good Option?

**DOI:** 10.3389/fneur.2021.632036

**Published:** 2021-02-22

**Authors:** Miguel Sáez-Alegre, Pablo García-Feijoo, Pablo Millán, Catalina Vivancos Sánchez, Víctor Rodríguez Domínguez, Jorge García Nerín, Alberto Isla Guerrero, María Luisa Gandía-González

**Affiliations:** ^1^Department of Neurosurgery, Hospital La Paz Madrid, Madrid, Spain; ^2^Department of Intensive Care Medicine, Hospital La Paz Madrid, Madrid, Spain; ^3^Hospital La Paz Institute for Health Research, Madrid, Spain; ^4^CranioSPain Research Group, Institute for Neuroscience and Sciences of the Movement, Autonomous University of Madrid, Madrid, Spain

**Keywords:** malignant stroke, large vessel occlusion, decompressive craniecotmy, middle cerebral artery, COVID-19

## Abstract

SARS-CoV2 infection can lead to a prothrombotic state. Large vessel occlusion, as well as malignant cerebral stroke have been described in COVID-19 patients. In the following months, given the increase in COVID-19 cases, an increase in malignant cerebral SARS-CoV2 associated strokes are expected. The baseline situation of the patients as well as the risk of evolution to a serious disease due to the virus, depict a unique scenario. Decompressive craniectomy is a life-saving procedure indicated in patients who suffer a malignant cerebral stroke; however, it is unclear whether the same eligibility criteria should be used for patients with COVID-19. To our knowledge seven cases of decompressive craniectomy and malignant cerebral stroke have been described to date. We report on a 39-year-old female with no major risk factors for cerebrovascular disease, apart from oral contraception, and mild COVID-19 symptoms who suffered from left hemispheric syndrome. The patient underwent endovascular treatment with stenting and afterward decompressive craniectomy due to a worsening neurological status with unilateral unreactive mydriasis. We present the case and provide a comprehensive review of the available literature related to the surgical treatment for COVID-19 associated malignant strokes, to establish whether the same eligibility criteria for non-COVID-19 associated strokes should be used. Eight patients, including our case, were surgically managed due to malignant cerebral stroke. Seven of these patients received decompressive craniectomy, and six of them met the eligibility criteria of the current stroke guidelines. The mortality rate was 33%, similar to that described in non-COVID-19 cases. Two patients had a left middle cerebral artery (MCA) and both survived after decompressive craniectomy. Our results support that decompressive craniectomy, using the current stroke guidelines, should be considered an effective life-saving treatment for COVID-19-related malignant cerebral strokes.

## Introduction

The World Health Organization declared COVID-19 a pandemic on 11 March 2020. Since then, health workers have modified the way they treat their patients facing new challenges, adapting to the lack of resources, coping with uncertainty and the emergence of COVID-19 related diseases (1).

A wide sample of neurological symptoms and SARS-CoV2 infection-associated diseases have already been described—large vessel strokes being one of the most life-threatening events related to COVID-19 (2, 3).

Ischemic cerebral stroke is one of the leading causes of disability and death worldwide, especially when large vessel occlusion is present. This lack of blood supply, especially in younger patients can lead to malignant cerebral edema, which can lead to transtentorial herniation. Decompressive craniectomy represents a life-saving treatment, reducing mortality in malignant cerebral infarction (4).

We present the case of a young, previously healthy, woman with mild COVID-19 who underwent a decompressive craniectomy due to a COVID19-related malignant stroke. Cases of this type of stroke may potentially increase in the following months, and at present, it is unclear whether the same eligibility criteria could be used in this new scenario.

We also review currently available literature of large vessel strokes in COVID-19 patients who underwent decompressive craniectomy to date, with a focus on surgery eligibility criteria, to try to conclude whether these criteria should be used in decision making in COVID-19 patients.

## Case Presentation

A 39-year-old healthy woman, G4P4, with mRS = 0, with a history of gestational diabetes and no major risk factors for cerebrovascular disease apart from taking oral contraception (desogestrel 75 mcg), was admitted after suffering a stroke while self-isolating at home for COVID-19. Familial history was not suggestive of a procoagulant state as no thrombotic or thromboembolic events were found in the patient's relatives. She did not smoke, drink alcohol, or take recreational drugs. She was married and unemployed and lived with her husband and his four children for whom she was the primary caregiver.

The patient contacted the health system for the first time because of a 4-day history of asthenia and ageusia. Chest radiography showed bilateral basal opacities and blood tests were normal except for a PCR of 73 mg/dl. Reverse-transcriptase polymerase chain (PCR) for SARS-CoV2 of a nasopharyngeal swab was positive. She was discharged and sent home with a diagnosis of mild bilateral SARS-CoV2 pneumonia.

On day 6 she was admitted to another hospital at 1:55 pm due to a low level of consciousness and right brachio-crural hemiparesis. Last seen, asymptomatic, at 5 am the day before, she was found unresponsive at 9 am by her husband. Global aphasia, right-side hemiplegia, right side sensory loss, gaze deviation toward the left side, and right homonymous hemianopsia were found. The National Institute Health Stroke Scale (NIHSS) scoring was 23 and no traumatic signs were found. The blood test showed LDH 293 UI/L, D-dimer 1,450 ng/mL, and C-reactive protein 17.5 mg/L. Simple head CT, CT perfusion scan, and angio-CT scans showed a left MCA and internal carotid artery (ICA) acute ischemic stroke (ASPECTS 5/10, TAN score 0-1) with 60% ischemic mismatch.

Due to lack of onset time, she was ineligible for thrombolysis but due to the perfusion scan findings, she was eligible for mechanical thrombectomy. She was transferred to our hospital, the on-call stroke center for that day, with the diagnosis of wake-up left MCA and ICA stroke in probable relation with COVID-19, arriving at 8:04 pm. At 9 pm, after obtaining consent from the patient's husband, she underwent a revascularization procedure. Left MCA recanalization was achieved. Distal unreachable thrombi in the left anterior cerebral artery (ACA) were observed, as well as a repletion defect in the extracranial internal carotid artery, suggestive of the presence of a floating thrombus or, less likely being an arterial dissection, needing the placement of a Roadsaver stent under lysine acetylsalicylate 500 mg. Double anti-aggregation was delayed until control head CT, scheduled 24 h later. The patient was admitted to the intensive care unit (ICU)

**Figure 1:**
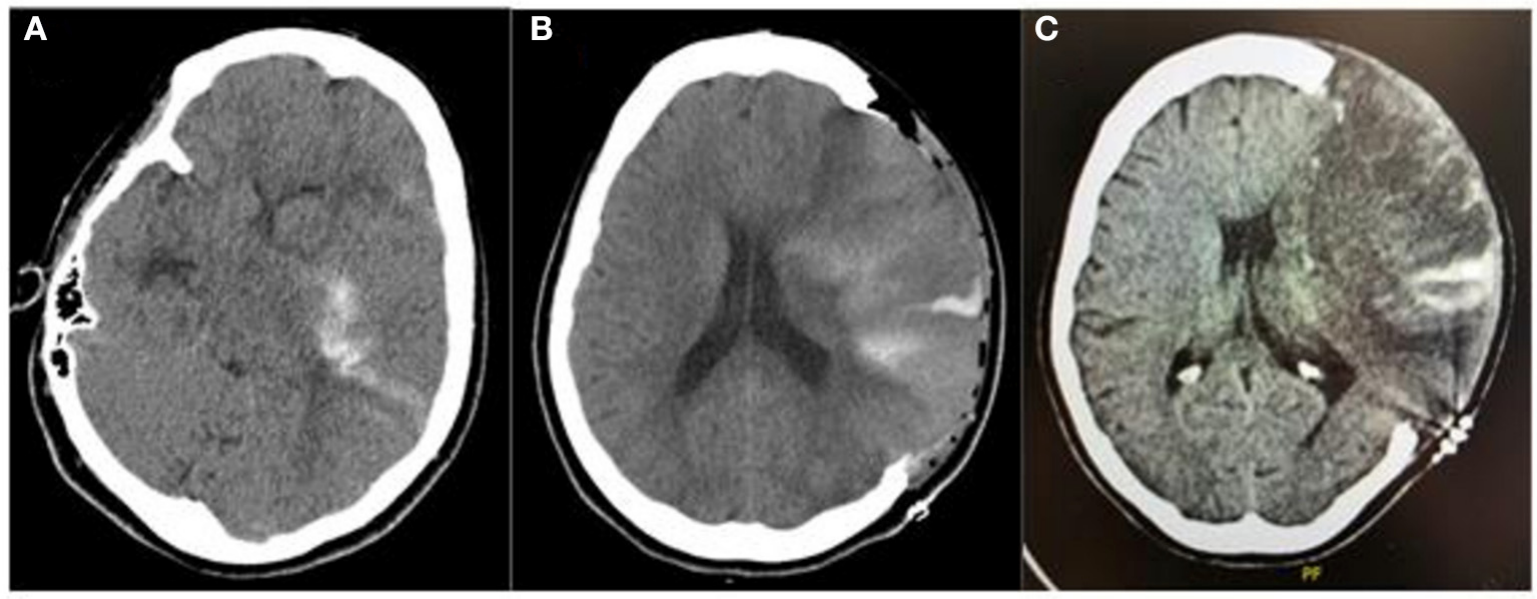
Pre- and post-craniectomy images: **(A)** Head CT scan at admission prior to surgery with left MCA and ICA acute ischemic stroke showing hemorrhagic transformation (PH-1); **(B)** Day 1 postoperative CT scan showing improvement in midline shift and cerebral edema with foci of hemorrhagic transformation, which remained stable; **(C)** Day 10 postoperative control CT scan.

**Figure 2:**
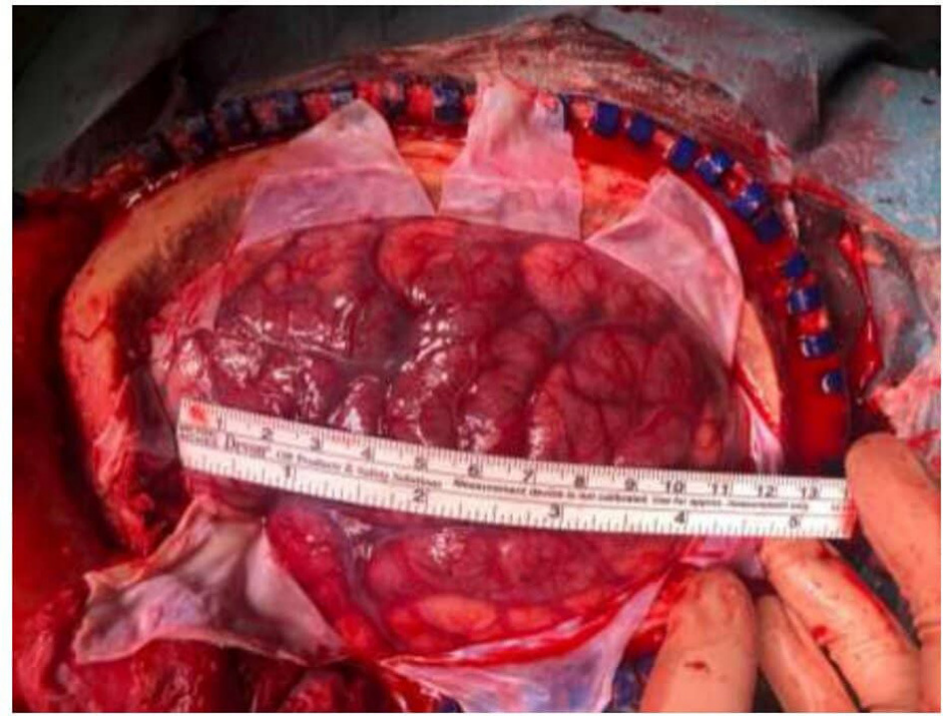
Intraoperative image after decompression, showing a 13 × 10 cm (antero- posterior and cranio-caudal length) left hemicraniectomy. Congestive brain with herniating through the bony defect was appreciated.

**Figure 3:**
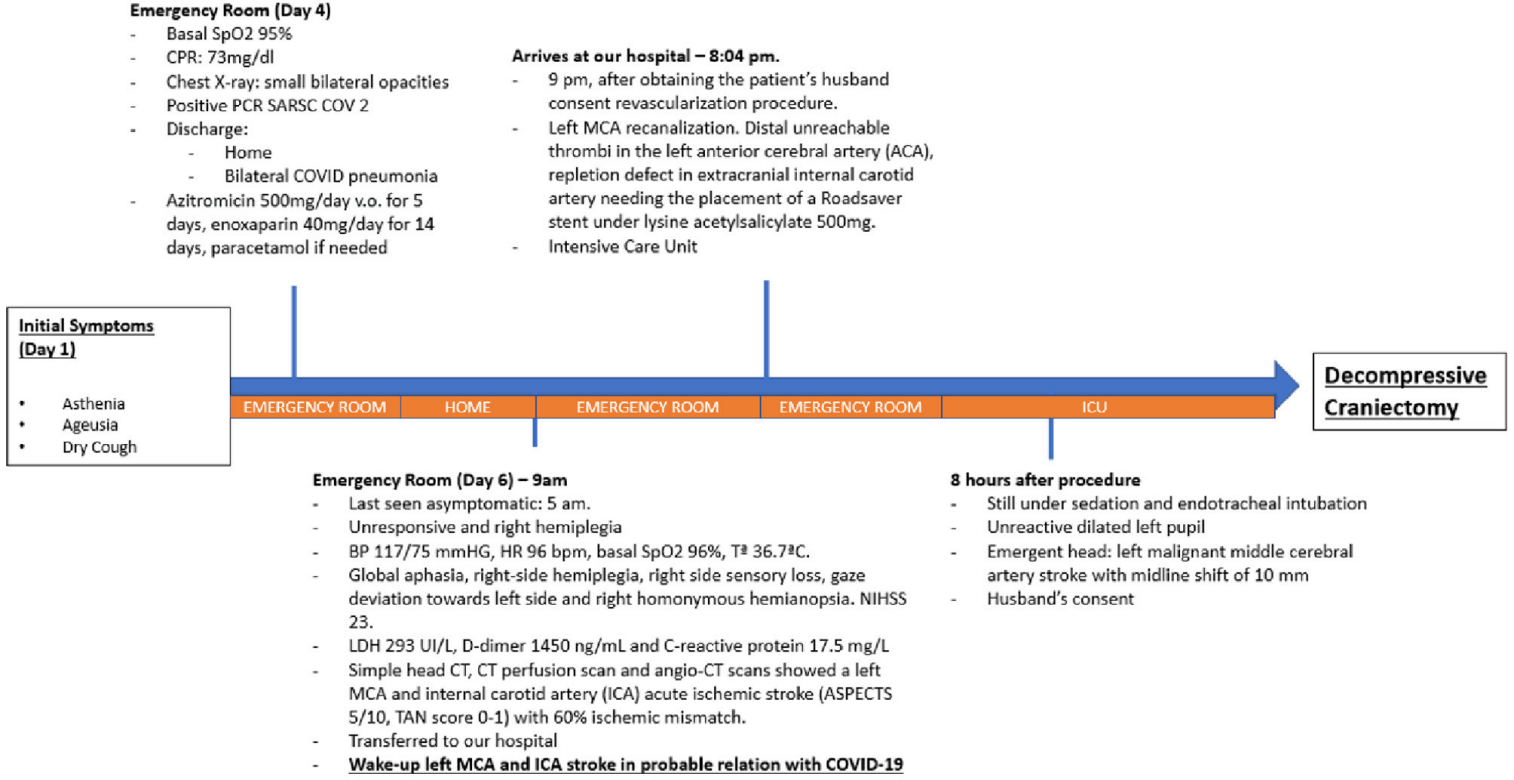
Figure showing patient's evolution from first symptoms to decompressive craniectomy.

**Figure 4:**
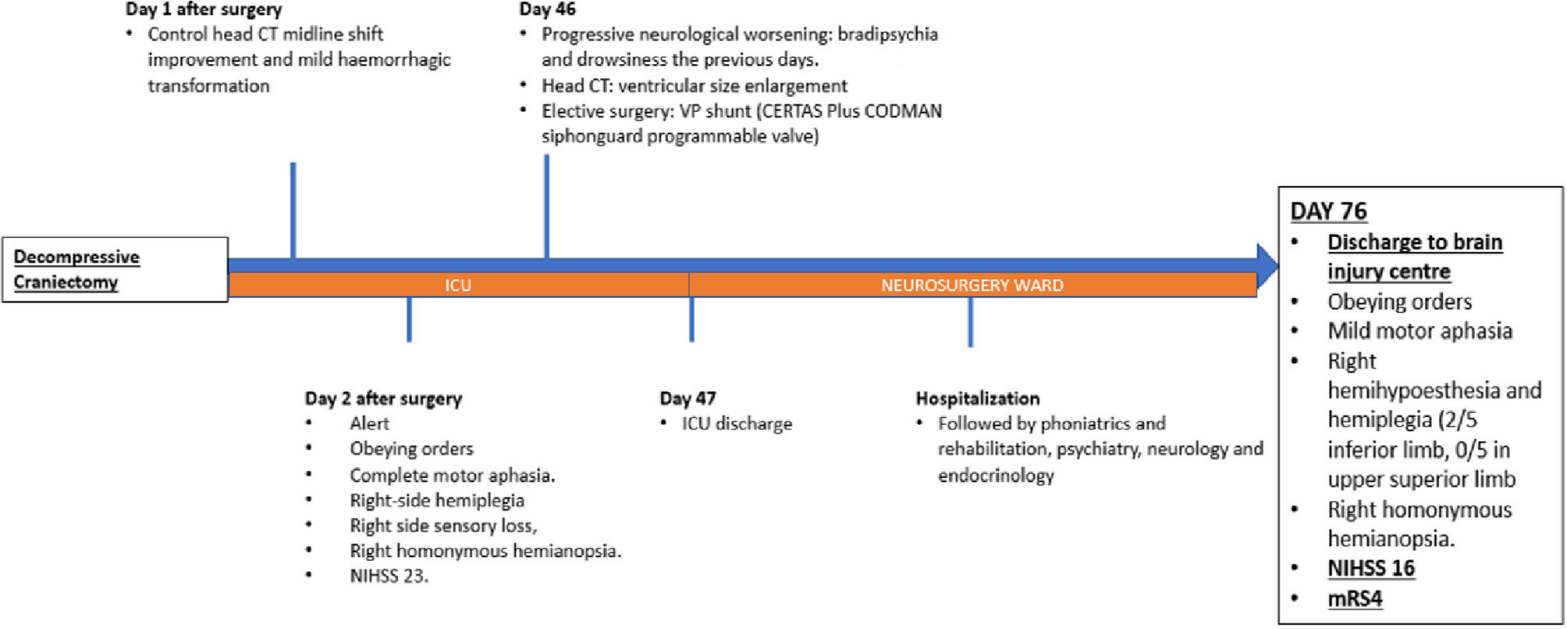
Figure showing patient's evolution from decompressive craniectomy to discharge.

Eight hours after the thrombectomy procedure and still, under sedation and endotracheal intubation, the patient developed an unreactive dilated left pupil. Emergent head CT scan showed a left malignant middle cerebral artery stroke with a midline shift of 10 mm and she therefore underwent a left decompressive craniectomy (Figures 1–4).

Postoperative day 1 control head CT showed midline shift improvement and mild hemorrhagic transformation. ASA 100 mg administration was started. Forty-eight hours after surgery, sedation was withdrawn. The patient was alert, obeyed simple and complex commands, seemed to understand her current situation but persisted total motor aphasia, right hemiplegia 0/5, and severe right side sensory loss.

Serial follow-up CT showed progressive ventricular size enlargement with progressive neurological worsening, consisting of bradypsychia and drowsiness. On postoperative day 46, a left-side ventriculoperitoneal shunt (CERTAS Plus CODMAN siphonguard programmable valve) was placed electively as part of our standard hydrocephalus treatment. The patient was finally discharged on postoperative day 76 to a brain injury center. Neurological status at discharge was assessed as a NIHSS 16 and mRS4. The patient was obeying orders, had mild motor aphasia, right hemihypoesthesia, and hemiplegia (2/5 grade of strength in right inferior limb and 0/5 in upper right superior limb using the Medical Research Council Muscle Testing Scale) and right homonymous hemianopsia. Treatment at discharge consisted of ASA 100 mg/day, enoxaparin 40 mg/day, sertraline 50 mg/day, and metformin 425 mg/12 h. The patient tolerated treatment and was adherent to treatment during admission. A new surgery to replace the bone flap will be discussed on an outpatient basis.

The patient's family was informed daily and they, as well as the patient, provided consent to the different interventions. For the emergency procedures (decompressive craniectomy and neurovascular revascularization), as the patient was unresponsive, verbal consent was obtained from the patient's husband. After the initial surgery, the patient was alert and consented to the ventriculoperitoneal shunt, the family was also informed and provided consent.

In the presence of negative neurological symptoms in a patient, a cranial CT scan should be performed urgently to rule out bleeding. In our patient's case, the CT scan already showed data of cerebral ischemia. Although the cases associated with COVID-19 have been described, the main causes of ictus, both frequent and infrequent, must be ruled out.

The pathogenesis of SARS-CoV2-associated neurologic symptoms has been intensively studied recently. It is reported that thromboembolic events are common in SARS-CoV2 infected patients, particularly large vessel occlusion strokes. In this patient, the main differential diagnosis is an alteration in coagulation or the presence of a DVT and a permeable oval foramen. Various tests were performed to try to clarify the etiology of the stroke. EKG and transthoracic echocardiogram were within normal limits and Doppler was not suggestive of a patent foramen ovale or an atrial septal defect. Procoagulant blood tests were normal and included antithrombin, free S-protein, activated protein C resistance, homocysteine, factor V gene mutation 61691A, prothrombin gene mutation G20120A, antiphospholipid antibodies, functional factor XII, and functional activity of protein C tests. Carotid Doppler showed no atheroma plaques with preserved and symmetrical flows. Left carotid stent was permeable with symmetrical velocities. Autoimmunity tests (ANA, antiDNAds, AntiCLP IgG and IgM, c and p ANCA) were negative. Serology for SARS-CoV2, 2 months after admission, showed positive IgG and IgM.

## Discussion

The COVID-19 pandemic represents a challenge for neurologists and neurosurgeons as large vessel strokes have been associated with SARS-CoV2 disease. The question of whether our protocols for stroke management are still valid or if they should be changed, remain (5).

Our institution has been one of the hardest hit during the pandemic (6). Spain has received an important number of patients and in particular, its capital, Madrid, and more specifically our hospital has been one of the world's focal points of the pandemic, mainly in the so-called first wave (7). Despite this, we had not had to deal with a similar clinical situation.

We present the clinical outcome of a young COVID-19 stroke patient who suffered only mild COVID-19 symptoms. We followed the usual stroke protocol at our hospital, based on international consensus guidelines (7), performing a decompressive craniectomy with dural expansion if a patient deteriorates neurologically within 48 h despite medical therapy. We discuss whether AHA/ASA 2018 Stroke Guidelines should also be followed in patients with COVID-19.

SARS-CoV2-associated neurologic symptom pathogenesis has been intensively studied. Thromboembolic events are common in SARS-CoV2 infected patients, particularly large vessel occlusion strokes (8).

Indeed, Kihira et al. (9) reported 2.4 times more risk of large vessel strokes in COVID-19 patients than in patients without COVID-19 infection. This association was not reported for small vessel strokes. They also reported on 62% of strokes involving M1-M2. This represents a risk factor to developing malignant cerebral edema. In our case the stroke was probably originally located in the left ACI and it then migrated to ipsilateral MCA and ACA. This MCA involvement was key to the development of cerebral edema.

COVID-19 patients have been shown to suffer a hypercoagulable state, increasing the risk of acute ischemic strokes, probably because some procoagulant and inflammatory pathways are activated. Mahboob et al. (10) suggested that antibody screening and immediate prophylactic anticoagulation may have reduced the risk of those events. In our case however, we did not find major risk factors for cerebrovascular disease, and antiphospholipid antibodies and the rest of the procoagulant tests were negative. Furthermore, prophylactic enoxaparin treatment was also started before the stroke was diagnosed, as mandated by the current guidelines (11). Our patient was under oral contraception, consisting of desogestrel, a progestogen that has been shown to increase the risk of venous thromboembolism (12).

In relationship to other risk factors, our patient was young and had no major risk factors. In contrast with this, Li et al. (13) in a single-center study reported that 5% of COVID-19 hospitalized patients suffered an acute ischemic stroke and that a higher incidence was seen in older patients with stroke risk factors. There is a strong association between stroke and major risk factors in COVID-19 patients, mainly age. Briefly, Rothstein et al. (14) reviewed a large series of patients with COVID-19 and acute cerebrovascular events, reporting that 95% of patients had a previous history of hypertension, 60% had a history of diabetes mellitus, and newly positive antiphospholipid antibodies were found in 75% of patients.

It is well-known that the prognosis of patients with malignant middle cerebral artery infarction is poor, with a mortality of up to 80% when applying the best medical care (15). Moreover, as recent studies show, the association between SARS-CoV2 and stroke worsens the prognosis and has been shown to increase the mortality rate associated with stroke (16), and also worsens the management of the disease and delays medical care (17).

Although it has been described as reducing mortality and morbidity (18–23) sometimes the baseline situation of the patient, the presence of antiaggregant and anticoagulant drugs, or severe sequelae, makes it difficult to take this decision. Another special situation is the involvement of the dominant hemisphere in the infarction. Neurosurgeons have to keep special considerations in mind as global aphasia is considered to be extremely disabling, with some patients reporting at discharge that they would have preferred not to have been operated on. However, recent studies showed similar clinical outcomes at medium- and long-term follow-up and up to 55% of patients with aphasia can partially recover (24, 25). This decision should be individualized and different factors, like the patient's willingness, timing, and familial situation should be considered.

In our case, the patient met our eligibility criteria and although she was under acetylsalicylate 500 mg, and although we knew that COVID-19 could worsen the prognosis, we indicated surgery. She had no previous wills, and we contacted her family which, after discussing treatment options and prognosis, provided consent to perform the surgery.

Age is an important factor. The benefit of decompressive craniectomy after malignant MCA infarction in reducing the outcome of death, without increasing the risk if severe disability, and while also increasing independence, has been demonstrated in patients under 60 years of age (7).

We reviewed the current literature and only eight cases (Table 1), four female and four male, have been surgically managed due to malignant cerebral strokes that have been reported to date, including our case.

**Table 1:** Covid19 related strokes, that required surgical treatment, described in the current medical literature.

	**Authors and Year**	**No. patients**	**Vessel**	**Age**	**Sex**	**DT until surgery**	**Major risk factors**	**Outcomes**
1	Liang et al. (2020) (18)	3	L ICA	57	F	<48H	HT, DM	Death
			R ICA	61	M	<24H	DM	Discharged
			L MCA	41	M	<24H	DM	Discharged
2	Roy et al. (2020) (19)	1	R ACA-MCA	46	F	9H	DM	Death
3	Rascón-Ramírez et al. (2020) (20)	2	L VA	35	M	<24H	None	Discharged
			R MCA	51	M	<24H	N/A	ICU
4	Alkhaibary et al. (2020) (21)	1	L CCA	31	F	72H	HT	ICU
5	Sáez-Alegre et al. (2020)	1	L MCA	39	F	8H	COCP	Discharged

*ACA, anterior cerebral artery; CCA, common carotid artery; COCP, combined oral contraceptive pill; DM, diabetes mellitus; DT, deterioration time; HT, hypertension; ICA, internal carotid artery; ICU, Intensive Care Unit; L, left; MCA, middle cerebral artery; R, right; VA, vertebral artery*.

In our case, surgery was performed 9 h after deterioration and there were no complications associated with COVID-19. Liang et al. (18) described three cases, one of them underwent surgery on day 2 post-stroke and died due to concomitant ST-segment–elevation myocardial infarction. Roy et al. (19) described a case who received surgical treatment 9 h after the stroke, unfortunately, the patient developed refractory hypoxemia in the postoperative period due to extensive pulmonary embolus and died. Alkhaibary et al. (20) described a case presenting with a left common carotid artery complete occlusion and underwent surgery at least 72 h after worsening. All cases except the one presented by Alkhaibary et al. (20) were treated according to the AHA/ASA 2018 Stroke Guidelines (48 h). In our case, although the patient was admitted to the hospital after 3 days of a low level of consciousness, we support the decision of the surgery as the patient was a 31-year-old previously healthy woman.

Our patient presented hydrocephalus. Rascón-Ramírez et al. (21) described two cases, one of them a 35-year-old man with right vertebral artery hypoplasia and anatomical absence of both posterior communicating cerebral arteries that suffered a partial occlusion of the left vertebral artery and underwent extensive suboccipital craniectomy and placing of an external ventricular drainage. However, this case differs from ours. The physiopathology of hydrocephalus due to the mass effect in the posterior fossa, such as the Rascón-Ramírez et al. case, is different from post-craniectomy hydrocephalus as in our case. This allowed us to perform elective surgery, placing a ventricular peritoneal shunt valve instead of an urgent external ventricular drain.

The mean age of patients with malignant middle cerebral artery stroke ranges from 43.5 to 63.5 years (26). In our study, the mean age was 45.1 years. The mortality rate among the general population who underwent decompressive craniectomy for malignant middle cerebral artery stroke, despite receiving the best treatment, ranges from 20 to 55% in recent series (22, 27). In our review, two of the eight cases are still in ICU, two died and four have been discharged, leaving a 33% mortality rate (2/6). This analysis includes patients not only with MCA infarction but also with the involvement of other vessels. If we look only at the presence of MCA involvement, four patients remain. One of the patients was still in the ICU at the time of publication of the case, and one of the three remaining cases died, leaving the mortality rate at 33%.

Although mortality seems to be the same, it is important to mention that in all cases, mortality was essentially derived from the severe COVID-19-related comorbidities previously detailed. Therefore, we can conclude that although further studies with a large series of patients are needed to achieve statistically significant results on COVID-19, it seems that large vessel occlusion related to COVID-19 occurs in younger patients and that surgery should be indicated following the same criteria as the pre-COVID-19 era.

## Conclusion

Our experience and our comprehensive literature review support decompressive craniectomy as a life-saving and effective treatment for patients with COVID-19 and malignant cerebral infarction. We suggest that surgery should be indicated following the same criteria as in the pre-COVID-19 era.

## Data Availability Statement

The original contributions presented in the study are included in the article/Supplementary Material, further inquiries can be directed to the corresponding author/s.

## Ethics Statement

Written informed consent was obtained from the patient for the publication of any potentially identifiable images or data included in this article.

## Author Contributions

MS-A, PG-F, and MG-G wrote the manuscript. CV, VR, JG, and PM helped gathering information. AI helped reviewing the manuscript. All authors contributed to the article and approved the submitted version.

## Conflict of Interest

The authors declare that the research was conducted in the absence of any commercial or financial relationships that could be construed as a potential conflict of interest.
